# Design of a green ammonia production process by machine learning

**DOI:** 10.1007/s44211-026-00903-3

**Published:** 2026-04-06

**Authors:** Sho Takaoka, Hiromasa Kaneko

**Affiliations:** Department of Applied Chemistry, School of Science and Technology, Meiji University, 1-1-1 Higashi-Mita, Tama-ku, Kawasaki, 214-8571 Kanagawa Japan

**Keywords:** Green ammonia, Bayesian optimization, Adaptive design of experiments, Machine learning, Process optimization

## Abstract

Green ammonia is attracting attention as a way of realizing a decarbonized society. In this study, we designed a green ammonia production process under limited power supply. In conventional process optimization, candidates for the design variables are selected based on the experience of the process engineer and calculation results, and optimization is performed by repeated simulations. However, as the number of design variables increases, an enormous number of simulations are required to search for optimal candidates. In this study, we used Bayesian optimization to select candidates for the design variables with a high probability of achieving the target values of the objective variables. Candidate selection and simulation were repeated to maximize green ammonia production with limited power supply. The process was compared with previously reported green ammonia production processes, which showed that the process designed in this study produced the maximum amount of green ammonia at ≤ 10 MW.

**Graphical abstract:**

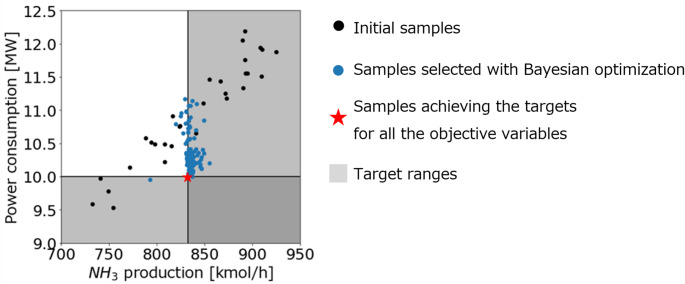

## Introduction

Ammonia is a useful raw material for nitrogen fertilizers, industrial refrigerants, and fuel for power generation and storage. Global ammonia production is approximately 150 million tons. An additional 2.3% annual increase in ammonia production is expected as the world’s population increases [1]. Many existing ammonia production processes use fossil fuels, such as natural gas as the feedstock, and the Haber–Bosch process is used for large-scale industrial production of ammonia [2, 3]. The reaction equations for ammonia synthesis using natural gas as the feedstock are as follows:

1 $$\:{\mathrm{C}\mathrm{H}}_{4}+2{\mathrm{H}}_{2}\mathrm{O}\to\:{\mathrm{C}\mathrm{O}}_{2}+4{\mathrm{H}}_{2}$$

2 $$\:4/7\cdot\:\mathrm{a}\mathrm{i}\mathrm{r}(1/2{\mathrm{O}}_{2}+2{\mathrm{N}}_{2})+4/7\cdot\:{\mathrm{H}}_{2}\to\:8/7\cdot\:{\mathrm{N}}_{2}+4/7\cdot\:{\mathrm{H}}_{2}\mathrm{O}$$

3 $$\:8/7\cdot\:{\mathrm{N}}_{2}+24/7\cdot\:{\mathrm{H}}_{2}\to\:16/7\cdot\:{\mathrm{N}\mathrm{H}}_{3}$$

The hydrogen and nitrogen necessary for ammonia synthesis are synthesized from methane, water, and air by Eqs. (1) and (2), and ammonia is synthesized by Eq. (3). The steam reforming reaction in Eq. (1) produces CO_2_ as a byproduct, along with hydrogen as the target product, so that approximately 1 mol of CO_2_ is emitted for every 2 moles of ammonia in the ammonia production process. In recent years, global warming caused by greenhouse gases, such as CO_2_, has become a serious concern, and it is estimated that ammonia production plants emit 1% of the CO_2_ emissions of the world. Therefore, a drastic reduction of greenhouse gas emissions is required [4]. Green ammonia is therefore attracting attention, and its research and development are progressing [5]. Green ammonia is ammonia that produces hydrogen and nitrogen from water electrolysis and air separation using electricity derived from renewable energy sources, such as wind and solar power, and thus no CO_2_ is emitted in the production process [4, 6]. Because the production of green ammonia uses electricity derived from renewable energy sources, it depends on land for energy resources and costs, the energy supply is unstable and small, and transportation costs are high. Therefore, decentralized and small-scale green ammonia production processes are attracting attention [7].

The design and optimization of the production processes of ammonia and green ammonia have been extensively investigated [8–10]. Babu et al. [11] modeled an ammonia synthesis reactor and optimized the reactor length using a quasi-Newton method. Carvalho et al. [12] used a combination of barrier functions with a direct search method to optimize the feed gas temperature and reaction pressure of an ammonia synthesis reactor. Cheema and Krewer [13] and Khademi and Sabbaghi [14] modeled and compared ammonia synthesis reactors with different cooling systems and numbers of reactor beds. Azarhoosh et al. [15] developed a mathematical model of an ammonia synthesis reactor and optimized the reactor temperature and feed flow rate using a genetic algorithm. Zhang et al. [16] compared the energy efficiencies of different hydrogen production methods for green ammonia production.

In the optimization of design variables, such as for ammonia production processes, it is impractical in terms of cost and time to rebuild the plant every time the values of the design variables are changed. Therefore, optimization using mathematical models and simulation software is performed in many cases. Frattini et al. [1] compared the costs of processes designed using Aspen Plus for different renewable energy sources. Sánchez and Martin [17] simulated a green ammonia production process and optimized the production conditions. In conventional process optimization, the candidates for the design variable are selected based on the experience of the process engineer and calculation results, and simulations are performed using the selected candidates for the design variable. If the simulation results do not meet the target values of the objective variables, such as the production volume and product yield, the candidate selection and simulation are repeated to improve the process. However, in design variable optimization, a large number of simulations are generally required to search for optimal candidates for the design variable because the search space becomes exponentially large as the number of design variables increases. For example, if there are 10 parameters for each of the six design variables, there are 1 million total combinations. If each simulation takes 1 h, it will take 1 million hours to complete all of the simulations, which is not realistic. In addition, if different processes are considered, the search space becomes even larger. Therefore, the processes need to be efficiently optimized.

This study focused on machine learning. The design of experiments (DoE) and the adaptive design of experiments (ADoE) are examples of methods for selecting the experimental conditions and candidates for the simulation by machine learning. The DoE determines the first candidate of the simulation among many candidates. The ADoE constructs a machine learning model based on the obtained simulation results and determines the next candidate using the estimates from the constructed model. The problem with the ADoE is that similar candidates tend to be selected as the next candidate. Therefore, Bayesian optimization (BO) was used in this study. BO can efficiently optimize design variables because it can select the next candidate for the simulation that has a high probability of achieving the goal. An example of the application of BO to process design is the study of the ethylene oxide production process, for which it has been confirmed that candidates for the design variable can be efficiently obtained and the method is reproducible [18].

Considering the above background, this study aims to design a green ammonia production process with limited power supply. BO was used to efficiently optimize the design variables X, which include physical parameters such as the feed rates of hydrogen and nitrogen, reactor inlet temperatures, reactor pressure, and conditions in the ammonia separation and refrigeration units. These variables directly affect both ammonia yield and power consumption. Detailed definitions and bounds of these variables are listed in Sect. "Results and discussion" (Table 1). AVEVA Process Simulation [19] is a process simulator that can switch between steady state and dynamic simulation modes. In this study, the process was designed with the process set to steady state simulation mode. Processes with different cooling systems and reactor beds for ammonia synthesis were designed and compared. The green ammonia production rate and power consumption obtained by optimization of X were compared with those of previously reported green ammonia production processes [7, 20–23].

Compared to previous studies that applied BO to general process optimization tasks, the novelty of this study lies in the integration of BO with a commercial simulator (AVEVA process simulation) for a power-constrained, small-scale green ammonia production process. We specifically explore six distinct process configurations with different cooling systems and reactor bed numbers, and apply a customized acquisition function to navigate trade-offs between ammonia production and power consumption. This enables the discovery of optimal conditions that maximize green ammonia yield under a strict energy constraint, a scenario not previously reported in the literature.

The present study should be regarded as a simulation-based process design and screening framework rather than as a direct plant-scale validation. Its practical value lies in efficiently narrowing down promising process configurations and operating conditions from a large design space under a unified set of simulation assumptions. The identified candidates can serve as a rational basis for subsequent pilot-scale studies, economic assessment, and system-level integration.

**Table 1:** X in the process with a four-bed adiabatic indirect cooling reactor

	X	Unit	Min	Max
X1	The feed of hydrogen	kmol/h	1100	1400
X2	The feed of nitrogen	kmol/h	0.332 × X1	0.336 × X1
X3	Pressure	bar	100	200
X4	1st stage reactor inlet temperature	K	673.15	723.15
X5	2nd stage reactor inlet temperature	K	673.15	723.15
X6	3rd stage reactor inlet temperature	K	673.15	723.15
X7	4th stage reactor inlet temperature	K	673.15	723.15
X8	Flash drum inlet temperature (Ammonia synthesis loop)	K	Refrigerant temperature + 5 K	Refrigerant temperature + 10 K
X9	Refrigerant section 1st flash drum inlet temperature	K	245.15	255.15
X10	Refrigeration compressor 3rd stage discharge pressure	bar	8.2	12.0
X11	Refrigeration compressor 2nd stage discharge pressure	bar	4.6	8.2
X12	Refrigeration compressor 1st stage discharge pressure	bar	1.013	4.6

## Methods

This study proposes an ADoE based on BO as an optimization method for X. BO uses Gaussian process regression (GPR) [24, 25], a regression analysis method, to construct a model and select new candidates for X.

### **Gaussian process regression**

GPR is a regression method in which, given an input X, the output y is a probability model following a normal distribution. Although GPR is a linear regression method, it can be extended to a nonlinear method by the kernel trick described below. Assuming that the GPR model is a linear model, the value of the objective variable for the *i*-th sample *y*^(*i*)^ is expressed as

4 $$\:{y}^{\left(i\right)}={\mathbf{x}}^{\left(i\right)}\mathbf{b}$$

where **x**^(*i*)^ is a vector of X for the *i*-th sample and **b** is the regression coefficient. If the distribution of **b** is normal with mean 0 and variance *σ*_b_^2^, *y*^(*i*)^ can be assumed to be normally distributed. The mean of *y*^(*i*)^ (*m*_*i*_) and covariance of *y*^(*i*)^ and *y*^(*j*)^ (*σ*_*yi*,*j*_^2^) are expressed as

5 $$\:{m}_{i}=\mathrm{E}\left[{y}^{\left(i\right)}\right]=\mathrm{E}\left[{\mathbf{x}}^{\left(i\right)}\mathbf{b}\right]={\mathbf{x}}^{\left(i\right)}\mathrm{E}\left[\mathbf{b}\right]=0$$

6 $$\begin{aligned} \sigma _{{yi,j}}^{2} = & {\mathrm{cov}}\left[ {y^{{\left( i \right)}} ,y^{{\left( j \right)}} } \right] = {\mathrm{cov}}\left[ {x^{{\left( i \right)}} b,x^{{\left( j \right)}} b} \right] \\ = & x^{{\left( i \right)}} {\mathrm{cov}}\left[ {b,b} \right]x^{{\left( j \right)}} = x^{{\left( i \right)}} x^{{\left( j \right)T}} \sigma _{b}^{2} \\ \end{aligned}$$

When **x**^(*i*)^ is transformed by a certain nonlinear function *φ*, the covariance *σ*_y*i*,*j*_^2^ is expressed as

7 $$\:{{\sigma\:}_{yi,j}}^{2}={\upphi\:}\left({\mathbf{x}}^{\left(i\right)}\right){\upphi\:}{\left({\mathbf{x}}^{\left(j\right)}\right)}^{\mathrm{T}}{{\sigma\:}_{\mathrm{b}}}^{2}=K\left({\mathbf{x}}^{\left(i\right)},{\mathbf{x}}^{\left(j\right)}\right)$$

where *K* is the kernel function. However, assuming that the value of *y*^(*i*)^ has a measurement error, the measurement error follows a normal distribution with mean 0 and variance *σ*_e_^2^, and the normal distribution of the measurement error for each sample is independent. The value of the objective variable *y*_obs_^(*i*)^ for the *i*-th sample including the measurement error is expressed as

8 $$\:{{y}_{\mathrm{o}\mathrm{b}\mathrm{s}}}^{\left(i\right)}={y}^{\left(i\right)}+{e}^{\left(i\right)}$$

Because *e*^(*i*)^ for each sample is independent, the covariance between *y*_obs_^(*i*)^ and *y*_obs_^(*j*)^ (*σ*_yobs*i*,*j*_^2^) is expressed as

9 $$\:{{\sigma\:}_{\mathrm{y}\mathrm{o}\mathrm{b}\mathrm{s}\:i,j}}^{2}=K\left({\mathbf{x}}^{\left(i\right)},{\mathbf{x}}^{\left(j\right)}\right)+{{\updelta\:}}_{i,j}{{\sigma\:}_{\mathrm{e}}}^{2}$$

When *i* = *j*, *δ*_*i, j*_ = 1, and otherwise *δ*_*i, j*_ = 0. If the number of samples for model construction is *n* and the design variable for the (*n* + 1)-th sample is **x**^(*n*+1)^, the (*n* + 1)-th sample can be estimated as a normal distribution with mean *m*(**x**^(*n*+1)^) and variance *σ*^2^(**x**^(*n*+1)^):

10 $$\:m\left({\mathbf{x}}^{(n+1)}\right)=k{\sum\:}_{n}^{-1}{\mathbf{y}}_{\mathrm{o}\mathrm{b}\mathrm{s}}$$

11 $$\:{{\upsigma\:}}^{2}\left({\mathbf{x}}^{(n+1)}\right)=K\left({\mathbf{x}}^{(n+1)},{\mathbf{x}}^{(n+1)}\right)+{{{\upsigma\:}}_{\mathrm{e}}}^{2}-k{\sum\:}_{n}^{-1}{k}^{\mathrm{T}}$$

*k* is expressed as

12 $$\:k=\left[K\left({\mathbf{x}}^{\left(1\right)},{\mathbf{x}}^{(n+1)}\right)\:\cdots\:\:K\left({\mathbf{x}}^{\left(i\right)},{\mathbf{x}}^{\left(n+1\right)}\right)\:\cdots\:K\left({\mathbf{x}}^{\left(n\right)},{\mathbf{x}}^{(n+1)}\right)\right]$$

The kernel functions of the GPR are shown below.

13 $$K\left( {{{\mathbf{x}}^{\left( i \right)}},{{\mathbf{x}}^{\left( j \right)}}} \right)={\theta _0}{{\mathbf{x}}^{\left( i \right)}}{{\mathbf{x}}^{\left( j \right)}}^{{\mathrm{T}}}+{\theta _1}$$

14 $$K\left( {{{\mathbf{x}}^{\left( i \right)}},{{\mathbf{x}}^{\left( j \right)}}} \right)={\theta _0}\exp \left\{ { - \frac{{{\theta _1}}}{2}{{\left\| {{{\mathbf{x}}^{(i)}} - {{\mathbf{x}}^{(j)}}} \right\|}^2}} \right\}+{\theta _2}$$

15 $$K\left( {{{\mathbf{x}}^{\left( i \right)}},{{\mathbf{x}}^{\left( j \right)}}} \right)={\theta _0}\exp \left\{ { - \frac{{{\theta _1}}}{2}{{\left\| {{{\mathbf{x}}^{(i)}} - {{\mathbf{x}}^{(j)}}} \right\|}^2}} \right\}+{\theta _2}+{\theta _3}{{\mathbf{x}}^{\left( i \right)}}{{\mathbf{x}}^{\left( j \right)}}^{{\mathrm{T}}}$$

16 $$K\left( {{{\mathbf{x}}^{\left( i \right)}},{{\mathbf{x}}^{\left( j \right)}}} \right)={\theta _0}\exp \left\{ { - \frac{1}{2}\sum\limits_{{k=1}}^{m} {{\theta _{1,k}}{{\left( {{x_k}^{{(i)}} - {x_k}^{{(j)}}} \right)}^2}} } \right\}+{\theta _2}$$

17 $$K\left( {{{\mathbf{x}}^{\left( i \right)}},{{\mathbf{x}}^{\left( j \right)}}} \right)={\theta _0}\exp \left\{ { - \frac{1}{2}\sum\limits_{{k=1}}^{m} {{\theta _{2,k}}{{\left( {{x_k}^{{(i)}} - {x_k}^{{(j)}}} \right)}^2}} } \right\}+{\theta _2}+{\theta _3}{{\mathbf{x}}^{\left( i \right)}}{{\mathbf{x}}^{\left( j \right)}}^{{\mathrm{T}}}$$

18 $$K\left( {{{\mathbf{x}}^{\left( i \right)}},{{\mathbf{x}}^{\left( j \right)}}} \right)={\theta _0}\left( {1+\frac{{\sqrt 3 {d_{i,j}}}}{{{\theta _1}}}} \right)\exp \left( { - \frac{{\sqrt 3 {d_{i,j}}}}{{{\theta _1}}}} \right)+{\theta _2}$$

19 $$K\left( {{{\mathbf{x}}^{\left( i \right)}},{{\mathbf{x}}^{\left( j \right)}}} \right)={\theta _0}\left( {1+\frac{{\sqrt 3 {d_{i,j}}}}{{{\theta _1}}}} \right)\exp \left( { - \frac{{\sqrt 3 {d_{i,j}}}}{{{\theta _1}}}} \right)+{\theta _2}+{\theta _3}{{\mathbf{x}}^{\left( i \right)}}{{\mathbf{x}}^{\left( j \right)}}^{{\mathrm{T}}}$$

20 $$K\left( {{{\mathbf{x}}^{\left( i \right)}},{{\mathbf{x}}^{\left( j \right)}}} \right)={\theta _0}\exp \left( { - \frac{{{d_{i,j}}}}{{{\theta _1}}}} \right)+{\theta _2}$$

21 $$K\left( {{{\mathbf{x}}^{\left( i \right)}},{{\mathbf{x}}^{\left( j \right)}}} \right)={\theta _0}\exp \left( { - \frac{{{d_{i,j}}}}{{{\theta _1}}}} \right)+{\theta _2}+{\theta _3}{{\mathbf{x}}^{\left( i \right)}}{{\mathbf{x}}^{\left( j \right)}}^{{\mathrm{T}}}$$

22 $$K\left( {{{\mathbf{x}}^{\left( i \right)}},{{\mathbf{x}}^{\left( j \right)}}} \right)={\theta _0}\left( {1+\frac{{\sqrt 5 {d_{i,j}}}}{{{\theta _1}}}+\frac{{5{d_{i,j}}^{2}}}{{3{\theta _1}^{2}}}} \right)\exp \left( { - \frac{{\sqrt 5 {d_{i,j}}}}{{{\theta _1}}}} \right)+{\theta _2}$$

23 $$K\left( {{{\mathbf{x}}^{\left( i \right)}},{{\mathbf{x}}^{\left( j \right)}}} \right)={\theta _0}\left( {1+\frac{{\sqrt 5 {d_{i,j}}}}{{{\theta _1}}}+\frac{{5{d_{i,j}}^{2}}}{{3{\theta _1}^{2}}}} \right)\exp \left( { - \frac{{\sqrt 5 {d_{i,j}}}}{{{\theta _1}}}} \right)+{\theta _2}+{\theta _3}{{\mathbf{x}}^{\left( i \right)}}{{\mathbf{x}}^{\left( j \right)}}^{{\mathrm{T}}},$$

where *x*_*k*_^(*i*)^ represents the value of the *k*th variable in the *i*th sample of X, *d*_*i*,*j*_ represents the Euclidean distance between **x**^(*i*)^ and **x**^(*j*)^, and *θ*_0_, *θ*_1_, *θ*_2_, *θ*_3_, *θ*_1,*k*_, and *θ*_2,*k*_ represent the hyperparameters. A machine learning model was constructed by selecting the kernel function with the best estimation performance by cross-validation among several kernel functions. The fold number for cross-validation was set to 10.

### **Design of experiments**

DoE is a method to select appropriate candidates from many candidates for X, such as the experimental conditions and simulation condition, to construct a good model. In this study, D-optimal design was used to select the first candidate. In D-optimal design, by maximizing the determinant of X-variables, which is called the D-optimal criterion, the amount of information obtained can be maximized and a good model can be constructed.

### **Adaptive design of experiments**

ADoE is a method in which experiments and simulations are performed using the first candidate selected by the DoE, and a search for the candidates for X is performed to ensure that the obtained experimental and simulation results achieve the target. In ADoE, a machine learning model is constructed from the values of y and X obtained from experiments and simulations with the selected candidates. The value of y can be estimated by inputting the candidates for X into the constructed machine learning model, and the candidates for X with a good estimate of y are selected as the next candidates. By repeating the above operations, an efficient candidate search can be performed.

### **Bayesian optimization**

BO is a powerful framework for global optimization of black-box functions that are expensive to evaluate, such as chemical process simulations. BO combines a probabilistic surrogate model with an acquisition function to determine the next point to evaluate, balancing exploration of uncertain regions and exploitation of promising candidates [26, 27].

In typical BO, a surrogate model—often a Gaussian Process Regression (GPR) model—is constructed from past observations to estimate the mean and uncertainty of the objective function. An *acquisition function* then evaluates candidate inputs based on the surrogate model, quantifying the expected benefit of sampling each candidate. Common acquisition functions include expected improvement, probability of improvement, and upper confidence bound.

BO is particularly well suited for chemical process design, where each simulation can be time-consuming, and the design space is high-dimensional. In this study, we applied BO using GPR as the surrogate model and a customized acquisition function based on the probability of satisfying target constraints (described later as PTR). The goal was to efficiently identify input conditions (design variables X) that maximize green ammonia production under a power consumption constraint.

**Fig. 1:**
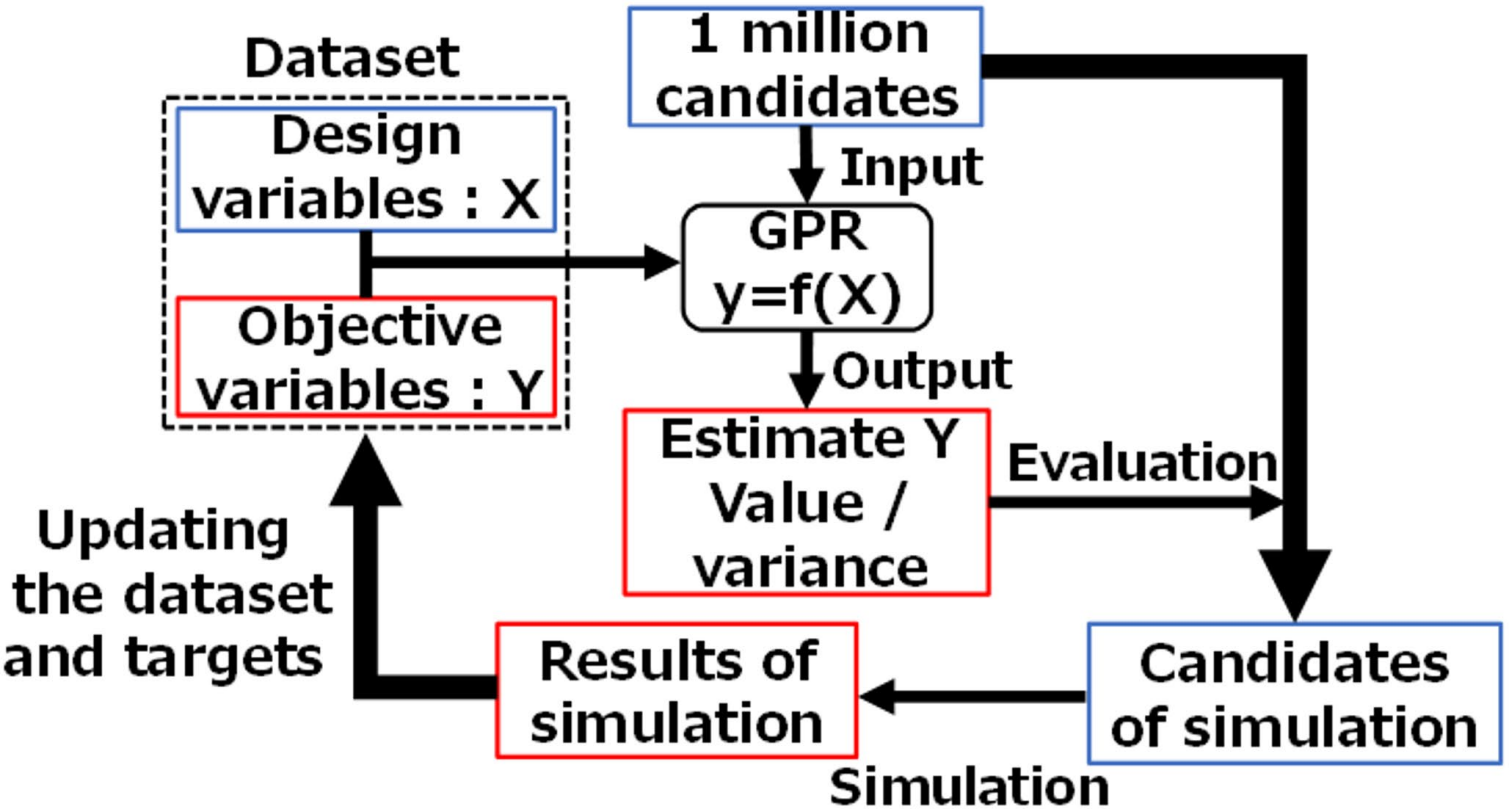
Outline of BO

In ADoE, where the candidate selection is based on the estimated value of y, there is a problem that if the candidates for X with good estimates of y are selected, similar candidates will continue to be selected and a large improvement in the value of y cannot be achieved. Therefore, in this study, the candidate selection for the design variable was carried out by BO, in which the candidate selection is based on the probability of achieving the target of y. A conceptual diagram of BO is shown in Fig. 1.

An overview of BO is given below. A model y = f(X) is constructed between X and y by GPR. One million candidates generated based on random numbers are input into the constructed model, and an estimate of y and the variance of y are output. The probability of achieving the target of y using the estimate of y and the variance of y is calculated. The candidate for X with the highest probability of achieving the ***y*** target is selected and simulations are run using the selected candidate. If the simulation results fail to achieve all of the y targets, the dataset is updated by adding the candidate for X and the simulation results to a new dataset, and model construction is performed again to select candidates. If the simulation results meet the targets for all y, the simulation is updated with a new target value of 1.05 times the green ammonia production rate that met the target, and the simulation is repeated up to the set maximum number of simulations. The factor of 1.05 was chosen to incrementally shift the target based on the previously achieved value, aiming to promote gradual exploration while avoiding abrupt changes that may hinder convergence. The updating of the target value is expected to maximize the green ammonia production rate under a limited electricity supply.

**Fig. 2:**
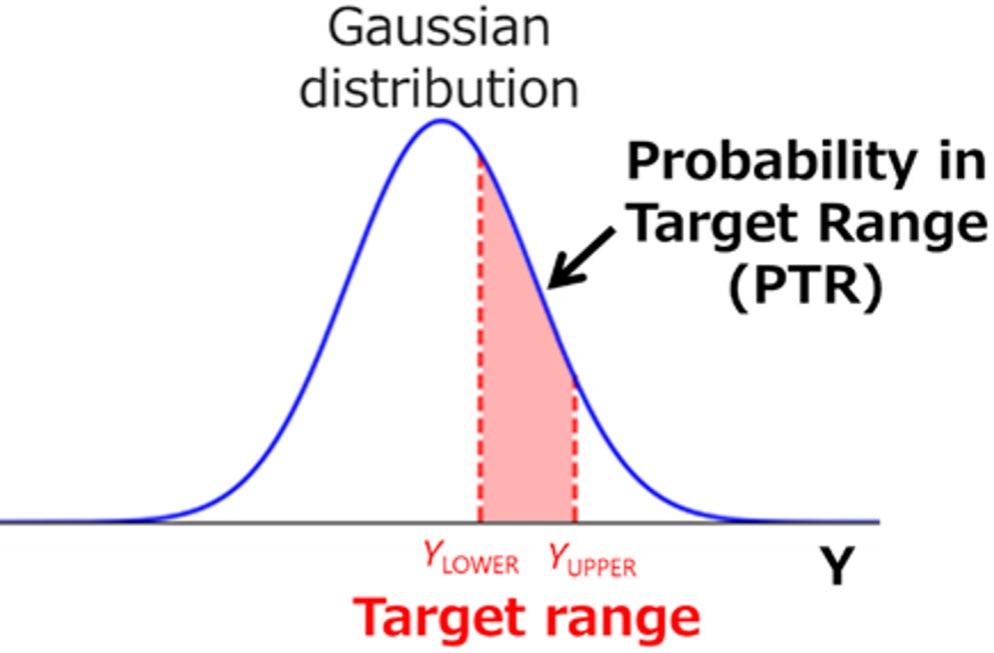
Outline of the PTR. The blue line is the Gaussian distribution of ***y***_**new**_^(*i*)^ with mean *m*(**x**_**new**_^(*i*)^) and variance *σ*^2^(**x**_**new**_^(*i*)^). The red region is the probability that the value of ***y*** falls within the target range

The probability in the target range (PTR), an acquisition function, was used as an evaluation index for the candidate selection in BO. The basic concept of PTR is shown in Fig. 2. The PTR is the probability that the value of the estimated ***y*** falls within the set target range (*y*_min_ ≤ y ≤ *y*_max_). When the new sample **x**_**new**_^(*i*)^ is input into the GPR model, it is assumed that *y*_new_^(*i*)^ is a normal distribution with mean *m*(**x**_**new**_^(*i*)^) and variance *σ*^2^(**x**_**new**_^(*i*)^). In this case, the probability PTR(**x**_**new**_^(*i*)^) can be obtained by integrating the normal distribution from *y*_min_ to *y*_max_ as follows:

24 $$\begin{aligned} \:{\mathrm{PTR}}\left( {{\mathbf{x}}_{{{\mathrm{new}}}} ^{{\left( i \right)}} } \right) & = \:\int \: _{{y\_min}}^{{y\_max}} \frac{1}{{\sqrt {2\pi \sigma ^{2} \left( {{\mathbf{x}}_{{{\mathrm{new}}}} ^{{\left( i \right)}} } \right)} }} \\ & = {\mathrm{exp}}\left\{ { - \frac{1}{{2\sigma ^{2} \left( {{\mathbf{x}}_{{{\mathrm{new}}}} ^{{\left( i \right)}} } \right)}}\left( {y - m\left( {{\mathbf{x}}_{{{\mathrm{new}}}} ^{{\left( i \right)}} } \right)} \right)^{2} } \right\}{\mathrm{d}}y \\ \end{aligned}$$

If there are multiple y-variables, the PTR is calculated for each y-variable. It is possible to search for X in which all of the y fall within the target range by summing the logarithmic values of the calculated PTR as the acquisition function PTR_all_(**x**_**new**_^(*i*)^):

25 $$\:{\mathrm{P}\mathrm{T}\mathrm{R}}_{\mathrm{a}\mathrm{l}\mathrm{l}}\left({{\mathbf{x}}_{\mathbf{n}\mathbf{e}\mathbf{w}}}^{\left(i\right)}\right)=\mathrm{log}\left({\mathrm{P}\mathrm{T}\mathrm{R}}_{1}\left({{\mathbf{x}}_{\mathbf{n}\mathbf{e}\mathbf{w}}}^{\left(i\right)}\right)+\cdots\:{+\:\mathrm{P}\mathrm{T}\mathrm{R}}_{n}\left({{\mathbf{x}}_{\mathbf{n}\mathbf{e}\mathbf{w}}}^{\left(i\right)}\right)\right)\:$$

The BO framework was implemented in Python and integrated with AVEVA process simulation for iterative optimization. At each iteration, the BO program proposes a new candidate set of X based on the current GPR model. The candidate is transferred to AVEVA process simulation, where steady-state simulations are executed using the specified process flowsheet. The simulation results—specifically, the green ammonia production rate and power consumption—are exported. Python then reads these outputs, updates the dataset, and retrains the GPR model to suggest the next candidate of X. This iterative loop continues, ensuring consistent and reproducible data transfer between both platforms.

### **Process configurations and simulation boundary**

**Fig. 3:**
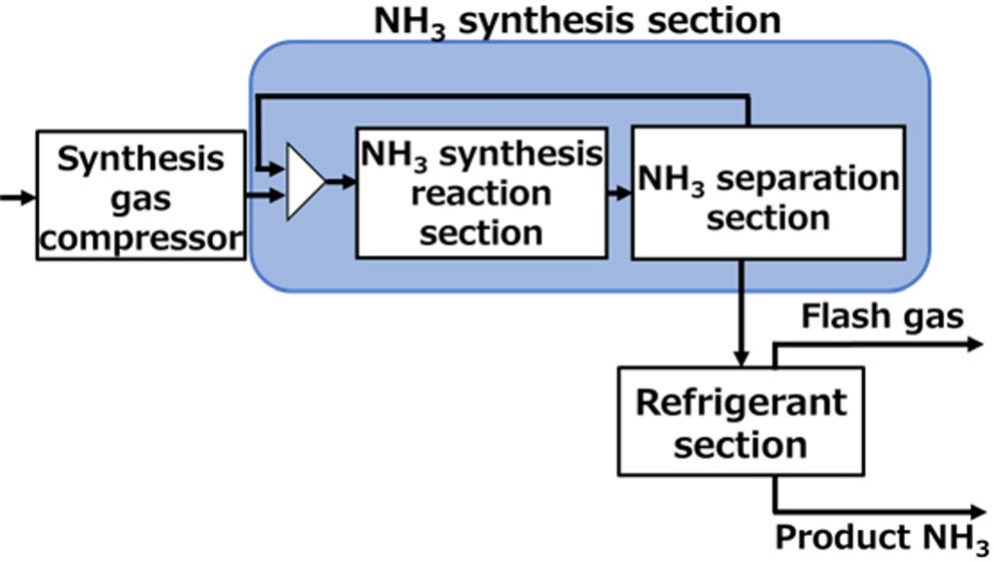
Schematic of the green ammonia production process

A block flow diagram of the designed green ammonia production process is shown in Fig. 3. The green ammonia production system consists of a synthesis gas compressor, an ammonia synthesis system with an ammonia synthesis reaction section and an ammonia separation section, and a refrigerant section. In the synthesis gas compressor, hydrogen and nitrogen, the raw materials, are pressurized to the ammonia synthesis pressure by a multi-stage compressor. Based on the Le Chatelier principle, the ammonia synthesis reaction under high ammonia synthesis pressure is considered to be the best way to increase ammonia production. However, increasing the synthesis pressure leads to an increase in the compressor power consumption. In the ammonia synthesis reaction section, ammonia is synthesized from the compressed synthesis gas using an ammonia synthesis reactor. Because ammonia synthesis is an exothermic reaction, lower reactor temperatures within the investigated range (673.15–723.15 K for the reactor inlet temperatures in this study) are favorable for the equilibrium conversion to ammonia. However, excessively low temperatures can reduce the reaction rate. Therefore, the reactor configuration must balance the thermodynamic advantage of lower temperature with sufficient reaction kinetics, which is one reason why multi-bed operation with intermediate cooling is important. In addition, the reaction is carried out in a multi-bed reactor because the ammonia concentration cannot be sufficiently increased in a one-bed reactor owing to equilibrium. Therefore, it is necessary to design the cooling system and the number of beds in the reactor. The outlet fluid of the ammonia synthesis reaction section contains unreacted hydrogen and nitrogen with ammonia. In the ammonia separation section, the fluid temperature is lowered using a refrigerant, and gas–liquid separation is performed using a flash drum. The gas fluid containing the unreacted gas is recycled and mixed with the synthesis gas. The refrigerant section circulates refrigerant for the condensation and separation of ammonia in the synthesis system and purifies the product, liquid ammonia.

The system boundary in this study was limited to the ammonia synthesis loop and its associated compression, separation, and refrigeration units. Upstream hydrogen production by water electrolysis and nitrogen supply by air separation were not explicitly included in the optimization. Therefore, the reported power consumption refers to the modeled process units within this boundary. Integration of upstream feedstock production is an important subject for future work.

## Results and discussion

In this study, six processes with different reactor cooling systems and numbers of reactor beds in the ammonia synthesis section (Fig. 3) were designed, and ***X*** were optimized for each process. Two reactor cooling systems, an adiabatic quench cooling reactor and an adiabatic indirect cooling reactor, which have been compared in several papers [16, 17], were used. The number of reactor beds was two to four. As a result of optimizing each of the six processes, the results are presented for the process using the four-bed adiabatic indirect cooling reactor that produced the largest amount of green ammonia at ≤ 10 MW.

X for the process using the four-bed adiabatic indirect cooling reactor contained 12 variables, and X and the range of candidates generated by random numbers are given in Table 1. y was set to two variables: the green ammonia production rate and power consumption (Table 2).

**Table 2:** y in the process with a four-bed adiabatic indirect cooling reactor

	y	Unit
y1	Green ammonia production	kmol/h
y2	Power consumption	MW

One million candidates for X were generated by random numbers, and 30 samples were selected as initial samples based on the D-optimal criterion. Simulations were performed using the 30 selected samples, and the value of y was obtained. The lower limit of the target range for the green ammonia production rate was set to 1.05 times the maximum green ammonia production rate in the simulation results with power use of ≤ 10 MW. The target was updated for each iteration of BO. The target range of the power consumption was set to ≤ 10 MW.

BO was performed for the process using a four-bed adiabatic indirect cooling reactor. The targets for all of the y were achieved in the 31st simulation (30 times D-optimal design and 1 times BO). After the targets were achieved, the lower limit of the target range for the green ammonia production rate was updated. After updating the target values, the targets for all of the objective variables were again achieved in the 136th simulation (30 times D-optimal design and 106 times BO). After further target value updates and BO, the 137th and subsequent simulations failed to find candidates for X that would achieve the targets. The results of the 136th simulation in which the targets for all of the y were achieved, as well as the values of the green ammonia production rate and power consumption before BO, are given in Table 3. As shown in Table 3, the optimized condition increased the green ammonia production rate from 754.34 to 831.69 kmol/h, while the power consumption increased from 9.5349 to 9.9939 MW. This increase in power consumption should not be interpreted as a deterioration in performance, because the optimization objective was to maximize green ammonia production under the constraint that the power consumption remain at or below 10 MW. Therefore, the optimized result indicates that the available power margin was effectively used to increase ammonia production while still satisfying the imposed power limit. It was confirmed that optimization of X increased the green ammonia production rate by 10%.

**Table 3:** Results of y that achieved the targets in the process with four-bed adiabatic indirect cooling

	Green ammonia production [kmol/h]	Power consumption [MW]
Before optimization	754.34	9.5349
After optimization	831.69	9.9939

**Fig. 4:**
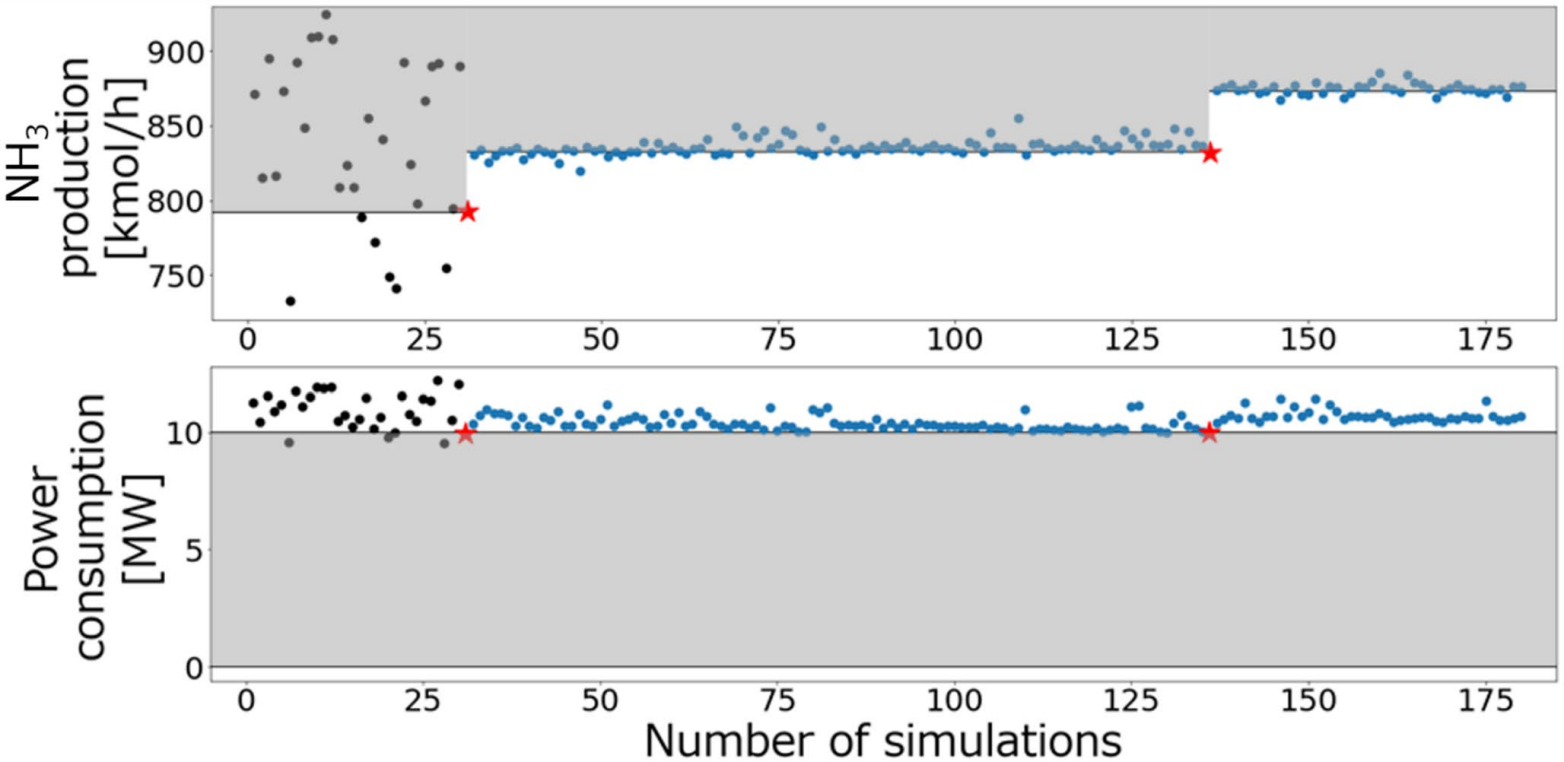
Results of 180 simulations with X selected by D-optimal design and BO for the green ammonia production rate (top) and power consumption (bottom) versus the number of simulations. The gray region, black points, blue points, and red points represent the target range, initial sample, sample selected by BO, and sample that achieved the targets for all of the objective variables, respectively

Plots of the number of simulation trials versus the green ammonia production rate and power consumption are shown in Fig. 4. The black points are the simulation results for X selected based on the D-optimal criterion, the blue points are the simulation results for X selected by BO, the red stars are the simulation results when the target values of all y were achieved, and the gray regions are the target ranges of y. The 31st simulation was useful because it provided the first feasible solution satisfying both objective-variable targets immediately after the initial D-optimal design stage. This early success suggests that the initial dataset was sufficiently informative for the GPR model to identify a promising candidate in the first BO iteration. After the 31st simulation, the lower limit of the green ammonia production target was updated to a stricter value, which made the simultaneous satisfaction of high production and power consumption ≤ 10 MW more difficult. As a result, some subsequent BO-selected candidates deviated from the target range, especially in terms of power consumption, before another target-achieving solution was found at the 136th simulation. In the 137th and subsequent simulations, it was not possible to find candidates that would achieve the targets for all of the y. The updated green ammonia production target was relatively high. Furthermore, there was a trade-off between the green ammonia production rate and power consumption. Therefore, it is considered that the target was not achieved because the results did not allow the power consumption to be less than 10 MW. X optimized for the process using a four-bed adiabatic indirect cooling reactor were determined by 30 iterations of D-optimal design and 106 iterations of BO. This means that the optimal X was found from 1 million candidates for X by 136 simulations, and it can be considered that the process was optimized efficiently.

**Fig. 5:**
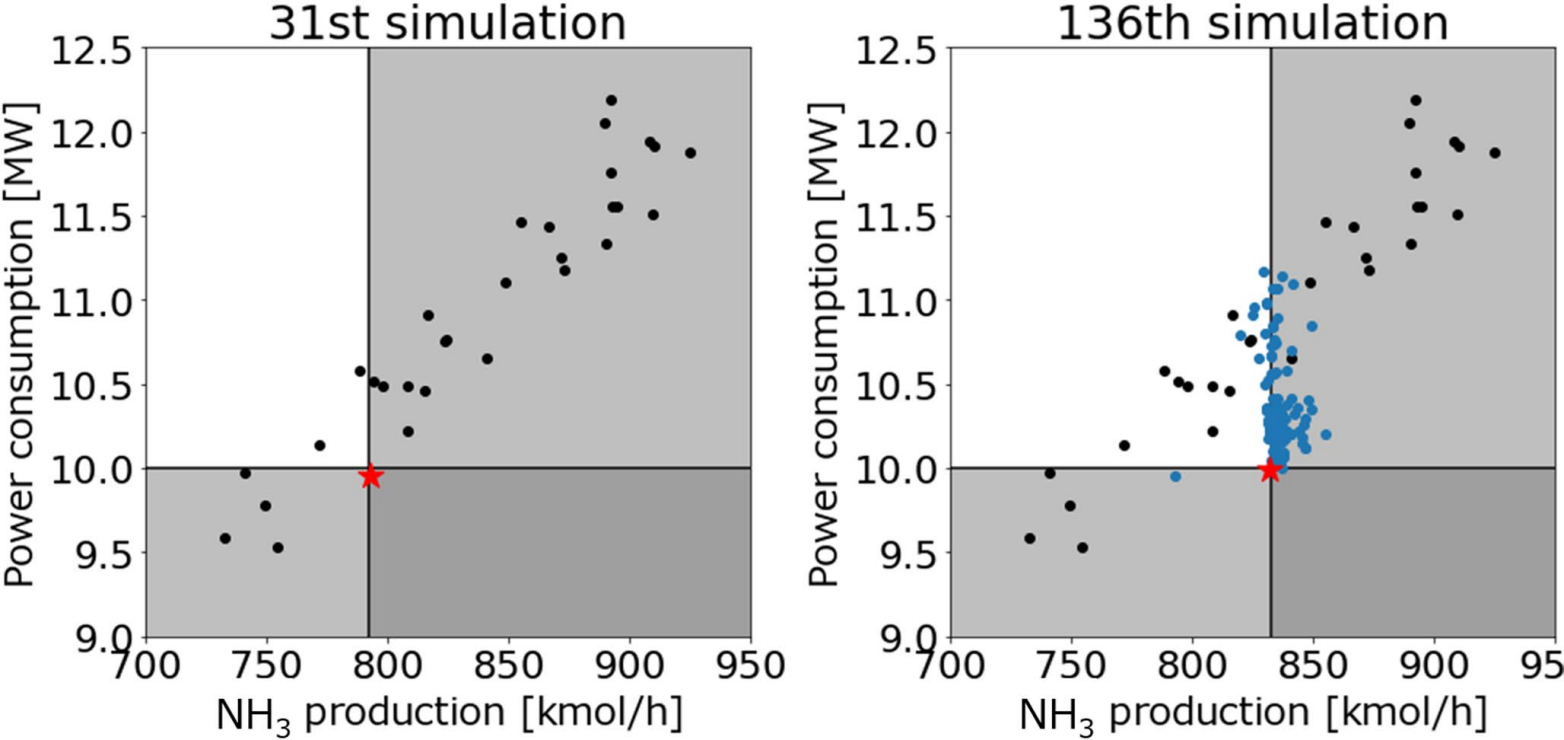
Plots of the power consumption versus the green ammonia production rate for up to the 31st and 136th simulations in which all of the objective variable targets were met. The gray region, black points, blue points, and red points represent the target range, initial sample, sample selected by BO, and sample that achieved the targets for all of the objective variables, respectively

The power consumption is plotted against the green ammonia production rate for the simulation results of the 31st and 136th runs in Fig. 5. As in Fig. 4, the black dots are the simulation results for X selected based on the D-optimal criterion, the blue dots are the simulation results for X selected by BO, and the red stars are the simulation results when the target values for all of the y were achieved. The gray regions are the target ranges of y. The black points in Fig. 5 indicate a trade-off between the green ammonia production rate and power consumption, making it difficult to increase production while maintaining the power consumption at or below 10 MW. The BO-selected blue points in the 136th-simulation plot are distributed closer to the boundary of the feasible region than the initial samples, indicating that BO progressively guided the search toward higher-production candidates under the power constraint. The red star represents the simulation result that simultaneously satisfied the updated production target and the power-consumption constraint.

**Fig. 6:**
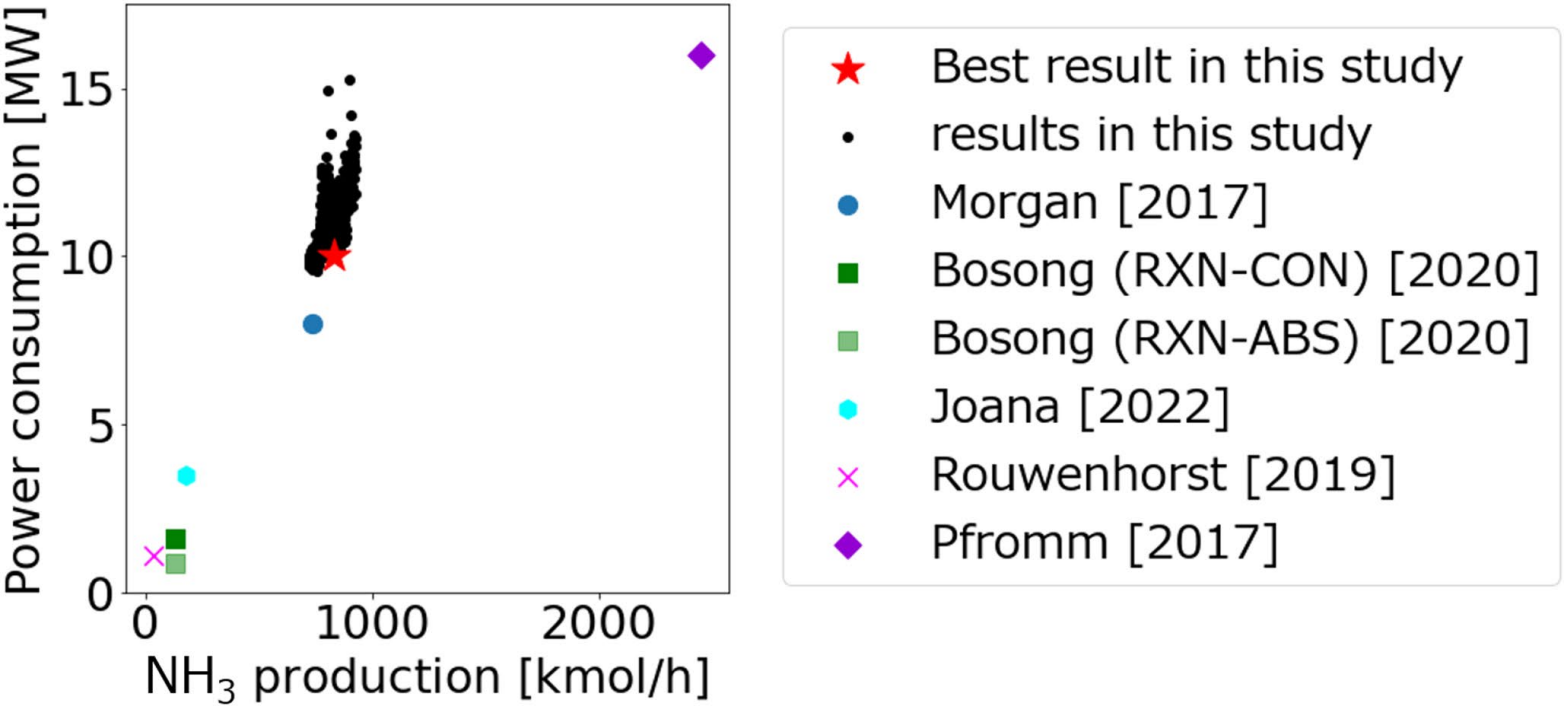
Comparison of the present results with those of previously reported green ammonia production processes

BO was performed for the other five processes in a similar manner. A plot of the green ammonia production rate versus the power consumption for all of the simulations and the results of previous studies of green ammonia production processes is shown in Fig. 6. The comparison in Fig. 6 should be interpreted with caution because the literature values were reported under different assumptions, scales, and system boundaries. Therefore, Fig. 6 is intended to provide a qualitative overview of the relative position of the present result around the 10 MW power range rather than a strictly homogeneous benchmark. Differences in reported ammonia production, including those in Pfromm [23], may arise from differences in process scale, feed conditions, and the inclusion or exclusion of auxiliary units, and thus are not directly attributable to the optimized variables considered in the present study alone. The simulation results obtained in this study showed the highest green ammonia production rate at 10 MW power consumption compared with previously reported green ammonia production processes.

## Conclusions

In this study, BO was used to efficiently search for candidates for X that meet the targets for the green ammonia production rate and power consumption. Comparison with previously reported green ammonia production processes confirmed that the process designed in this study has the maximum green ammonia production rate at ≤ 10 MW. Process optimization takes an enormous amount of time because optimization is performed for a large number of X. However, BO, in which candidates for X are selected based on the probability of achieving the target of y, can be used to efficiently optimize X, and thus cost and time reduction can be achieved in new process design. While the present study is based on steady-state simulations and direct plant-scale verification is beyond the scope of this work, the results provide a practically useful basis for screening promising green ammonia process configurations under power constraints. Future work should include integration of upstream feedstock production, pilot-scale validation, and evaluation of economic feasibility and operational robustness. In the future, integration of the feedstock production process and verification of the economics and robustness of the process will lead to early adoption of the green ammonia production process in societies.

## Data Availability

The datasets generated and analyzed during the current study, including the Bayesian optimization results and process simulation outputs, are available from the corresponding author on reasonable request.

## References

[1] D. Frattini, G. Cinti, G. Bidini, U. Desideri, R. Cioff, E. Jannelli, A system approach in energy evaluation of different renewable energies sources integration in ammonia production plants. Renew. Energy. **99**, 472–482 (2016)

[2] M. Farsi, N. Chabi, M.R. Rahimpour, Modeling and optimization of ammonia process: Effect of hydrogen unit performance on the ammonia yield. Int. J. Hydrog Energy. **46**, 39011–39022 (2021)

[3] C. Smith, A.K. Hill, L. Torrente-Murciano, Current and future role of Haber-Bosch ammonia in a carbon-free energy landscape. Energy Environ. Sci. **13**, 331–344 (2020)

[4] K. Smart, Review of Recent Progress in Green Ammonia Synthesis: Decarbonisation of fertiliser and fuels via green synthesis. Johns. Matthey Technol. Rev. **66**(3), 230–244 (2022)

[5] H. Ishaq, I. Dincer, Design and simulation of a new cascaded ammonia synthesis system driven by renewables. Sustainable Energy Technol. Assess. **40**, 100725 (2020)

[6] https://greenammonia.org/en

[7] B. Lin, T. Wiesner, M. Malmali, Performance of a Small-Scale Haber Process: A techno-economic analysis. ACS Sustainable Chem. Eng. **8**, 15517–15531 (2020)

[8] P. Lassak, J. Labovsky, L. Jelemensky, Influence of parameter uncertainty on modeling of industrial ammonia reactor for safety and operability analysis. J. Loss Prev. Process. Ind. **23**, 280–288 (2010)

[9] M. Aziz, T. Oda, A. Morihara, T. Kashiwagi, Combined nitrogen production, ammonia synthesis, and power generation for efficient hydrogen storage. Energy Procedia. **143**, 674–679 (2017)

[10] C. Quintero-Masselski, J.F. Portha, L. Falk, Conception and optimization of an ammonia synthesis superstructure for energy storage. Chem. Eng. Res. Des. **177**, 826–842 (2022)

[11] B.V. Babu, R. Angira, Optimal design of an auto-thermal ammonia synthesis reactor. Comput. Chem. Eng. **29**(5), 1041–1045 (2005)

[12] E.P. Carvalho, C. Borges, D. Andrade, J.Y. Yean, M.A.S.S. Ravagnani, Modeling and optimization of an ammonia reactor using a penalty-like method. Appl. Math. Comput. **237**, 330–339 (2014)

[13] I.I. Cheema, U. Krewer, Optimisation of the Autothermal NH3 Production Process for Power-to-Ammonia. Process. **8**(1), 38 (2020)

[14] M.H. Khademi, R.S. Sabbaghi, Comparison between three types of ammonia synthesis reactor configurations in terms of cooling methods. Chem. Eng. Res. Des. **128**, 306–317 (2018)

[15] M.J. Azarhoosh, F. Farivar, H.A. Ebrahim, Simulation and optimization of a horizontal ammonia synthesis reactor using genetic algorithm. RSC Adv. **4**(26), 13419–13429 (2014)

[16] H.F. Zhang, L.G. Wang, J.V. Herle, F. Marechal, U. Desideri, Techno-economic comparison of green ammonia production processes. Appl. Energy. **259**, 114135 (2020)

[17] A. Sanchez, M. Martin, Optimal renewable production of ammonia from water and air. J. Clean. **178**, 325–342 (2018)

[18] R. Iwama, H. Kaneko, 2021. Design of ethylene oxide production process based on adaptive design of experiments and Bayesian optimization. J. Adv. Manuf. Process., **3**(3), e10085

[19] https://www.aveva.com/en/products/process-simulation/

[20] E.R. Morgan, J.F. Manwell, J.G. McGowan, Sustainable Ammonia Production from US Offshore Wind Farms: A Techno-Economic Review. ACS Sustainable Chem. Eng. **5**(11), 9554–9567 (2017)

[21] J. Sousa, W. Waiblinger, K.A. Friedich, Techno-economic Study of an Electrolysis-Based Green Ammonia Production Plant. Ind. Eng. Chem. Res. **61**, 14515–14530 (2022)

[22] K.H.R. Rouwenhorst, Van der A.G.J. Ham, G. Mul, S.R.A. Kersten, Islanded ammonia power systems: Technology review & conceptual process design. Renew. Sustainable Energy Rev. **114**, 109339 (2019)

[23] P.H. Pfromm, Towards sustainable agriculture: Fossil-free ammonia. J. Renew. Sustainable Energy. **9**(3), 034702 (2017)

[24] C.E. Rasmussen, C.K. Williams, *I. 2005. Gaussian processes for machine learning*., MIT Press, MA

[25] https://scikit-learn.org/stable/modules/generated/sklearn.gaussian_process.GaussianProcessRegressor.html

[26] B. Shahriari, K. Swersky, Z. Wang, Ryan P. Adams, and Nando De Freitas. Taking the human out of the loop: a review of Bayesian optimization. Proceedings of the IEEE 104, no. 1 (2015): 148–175

[27] S. Tao, A. Van Beek, D.W. Apley, W. Chen, Multi-model Bayesian optimization for simulation-based design. J. Mech. Des. **143**(11), 111701 (2021)

